# The overlooked role of the home in mass shooting fatalities

**DOI:** 10.1186/s40621-025-00602-z

**Published:** 2025-07-21

**Authors:** Wilson H. Hammett, Pragya Bhuwania, Jody Heymann

**Affiliations:** 1 Fielding School of Public Health, University of California, Los Angeles, 650 Charles E Young Dr S, Los Angeles, CA 90095-1772 USA; 2 WORLD Policy Analysis Center, UCLA Fielding School of Public Health, 621 Charles E. Young Drive South, 2225 Life Science Building, Los Angeles, CA 90095-1772 USA; 3 Luskin School of Public Affairs, University of California, Los Angeles, Los Angeles, CA USA

**Keywords:** Gun violence, Mass shootings, Domestic violence, Child and adolescent health, Gender

## Abstract

**Background:**

Prevention efforts for mass shooting fatalities often focus on public events, overlooking where most fatalities occur. This study analyzes a comprehensive database to better inform prevention strategies.

**Methods:**

Using data from the Gun Violence Archive (GVA), we identified 252 mass shooting events (2014–2023) with four or more deaths, excluding the perpetrator, resulting in 1,464 fatalities. A media analysis determined location and links to domestic violence. Mortality burden by location, age, gender, and domestic violence was calculated, with tests of association performed.

**Results:**

We found that half (50%) of mass shooting fatalities occurred in homes—five times more than in businesses/workplaces (10%). Among children (0–9), 89% of fatalities occurred in homes, compared to 62% among older children and teens (10–17) and 44% among adults (18+). Women were more likely to be killed in mass shootings at home (50%) than men (40%). Fatalities were disproportionately concentrated in homes across all age groups (*p* < 0.001). Domestic violence-related mass shootings accounted for 46% of all fatalities, with 70% of fatalities from domestic violence-related events occurring in homes.

**Conclusions:**

Public discourse neglects home-based mass shootings, which disproportionately affect women and children. Targeted interventions, especially addressing domestic violence, are critical for reducing fatalities.

**Supplementary Information:**

The online version contains supplementary material available at 10.1186/s40621-025-00602-z.

## Background

Despite constituting a small proportion of firearm fatalities each year [1], mass shootings in the United States (U.S.) have devastating consequences that extend far beyond the loss of life. Survivors, witnesses, and families of those injured or killed often experience severe trauma, including post-traumatic stress disorder (PTSD), increased suicide risk, and substance abuse [2–4]. Community members and those exposed to media coverage of mass violence events can also experience emotional distress [3, 5–8]. Nearly one-quarter of U.S. adults report altering their daily routines and one-third report avoiding certain locations due to fear of mass shooting incidents [9]. Mass shootings also play a central role in political debates around gun violence and potential solutions to reduce gun violence in the U.S [10, 11].

What constitutes a “mass shooting” has not been federally defined, and definitions used by databases, researchers, and media sources vary widely. Many focus on “public mass shootings” or define mass shootings as incidents with four or more fatalities occurring in public, populated areas, such as festivals, schools, or houses of worship [2, 12–15]. These definitions and the media focus on public mass shootings perpetrated by individuals unknown to the victims reinforce public perception that these types of events are the most common form of mass shooting and pose the greatest risk to public health. However, this way of framing mass shootings neglects those shootings that occur in private residences and may occur between family members or current or former romantic partners [10, 16].

Prior research has demonstrated a clear link between domestic violence and mass shooting events [12, 17, 18], and has explored the relative burden of “public” and “private” mass shootings [19–21], but has not looked in detail at the locations in which mass shooting fatalities occur, nor how the location of fatality may vary by age and gender. This study examines the burden of mass shootings across location type, determining the location(s) which may pose the most risk to public health, across age and gender groups. Policymakers and public health professionals must understand where mass shooting fatalities occur to develop effective solutions to reduce fatality. Understanding whether locations of fatalities vary by age group and gender can help target interventions more effectively.

## Methods

The overall aim of this study is threefold: to characterize the locations in which fatalities from mass shootings occurred most frequently between 2014 and 2023, to determine whether these locations differ significantly across age and gender groups, and to investigate the potential relationship between fatalities occurring in home-based shootings and those occurring within the context of domestic violence.

### Study sample

This study defines “mass shooting” as a shooting in which four or more people were killed, excluding the perpetrator (if the perpetrator died by suicide or was killed by law enforcement), in any circumstances or location, in rapid succession. Spree shootings that occurred in multiple locations, if they occurred in rapid succession and were carried out by the same shooter(s), and incidents with multiple shooters met inclusion criteria. We chose this definition to ensure consistency with prior literature [17]. This study analyzed a sample of all mass shootings that met the above inclusion criteria and occurred within the U.S. between January 1, 2014, and December 31, 2023, encompassing all 50 states and the District of Columbia. A total of 252 mass shootings met inclusion criteria, resulting in 1,464 fatalities.

### Data sources

This study utilized the Gun Violence Archive (GVA) mass shooting database (2014–2023) as the primary data source. GVA provides comprehensive data on mass shooting events, including date, location, fatalities, and injuries. While GVA includes a measure of “mass murder” that uses a definition aligned with our chosen definition of mass shooting, GVA has only used this measure since 2019. However, GVA’s mass shooting database includes five additional years of data (2014–2018). GVA defines “mass shooting” as any shooting event in which four or more individuals were shot (not necessarily killed) in rapid succession [22]. We restricted the mass shooting database to events with four or more fatalities, excluding the perpetrator, to generate our sample of events.

GVA was chosen as a data source because of its comprehensiveness and use in prior investigations of the relationship between domestic violence and mass shootings [17]. Unlike other databases, GVA encompasses incidents often overlooked by other sources, such as those classified as “gang-related,” “drug-related,” or occurring within domestic settings and provides data from all 50 states for all years since 2014, unlike the National Violent Death Reporting System (NVDRS) [23]. The database is compiled through a rigorous process involving automated queries and manual analysis of local and state police reports, media sources, and government records. Each entry undergoes a double-coding process and is linked to its original sources. For each event, GVA records demographic information about the fatalities, including age (or age group when exact age is unavailable), gender, and role (perpetrator or non-perpetrator). These data points were extracted for this analysis.

Additionally, for each event that met our inclusion criteria, we conducted a comprehensive Google search of news coverage of the event to determine additional details to supplement or confirm information available within the GVA database. Search terms included event address, event date, and perpetrator name (if known), Through a thorough reading of each article in totality, we determined whether each event was related to domestic violence (if the perpetrator had a familial or current or past intimate partner relationship with a victim), and demographic characteristics such as age and gender of fatality, and role of the fatality in the shooting (perpetrator or non-perpetrator). For larger profile shootings with higher fatality counts, up to 20 news articles were reviewed for each event, or until saturation was achieved. For smaller profile shootings that received limited media coverage, we reviewed all available news coverage.

### Study variables

We classified location of mass shooting events as occurring in a home if they took place exclusively within or immediately outside a residence, excluding spree shootings that occurred across multiple locations, regardless of whether the residence belonged to one or more of the victims. Separating “occurred in a home” and “domestic violence-related” in classifying events allows for an examination of overlap and differences of these categories, which are often conflated in discourse and research [24]. We classified other, non-home locations of mass shooting fatalities as: school, business/workplace, place of worship, festival/parade, bar, street, car, restaurant, and “other”, which combined all locations with *n* < 10 in the full sample of mass shooting fatalities (group home, airport, military base, hospital, shopping mall, campground, abandoned building, dance studio, motel, and non-residential farm).

We considered events as “domestic violence related” if any family member or current/former romantic partner was a victim, including children, siblings, or parents of the perpetrator. Since GVA does not include family members in its definition of “domestic violence”, we reviewed news articles to determine whether events fit this study’s definition. Fatalities from unsolved events in which perpetrator names were unknown made up 4% of all fatalities in this sample. For most unsolved events, media reports suggested robbery, gang violence, or drug involvement, indicating that it is unlikely that these events were domestic violence related. Therefore, we coded unsolved events as “non-domestic violence related” for this analysis. This choice provides a conservative estimate of the number of fatalities from domestic violence related events. To test the sensitivity of our results, we conducted additional analysis dropping fatalities from unsolved events (Appendix A).

### Analytical strategy

In Aim 1, we estimated the count and proportion of mass shooting fatalities that occurred across the study period (2014–2023) by event location for each of three age groups: children (ages 0–9), older children and teens (ages 10–17), and adults (ages 18+). Age groups under age 18 were separated into two distinct categories (0–9 years and 10–17 years) to reflect distinct developmental stages and corresponding exposure contexts. The 0–9 age group represents early to middle childhood, during which children are typically in settings with high levels of adult supervision (e.g., homes, daycare centers, or elementary schools). The 10–17 age group corresponds to late childhood and adolescence, in which youth begin to experience increased autonomy while remaining legally dependent on parents or guardians. This stratification allowed for separate analysis of fatalities based on developmental and environmental differences. We employed the Chi-square goodness-of-fit test to detect whether the proportion of fatalities varied significantly across locations for each age group. This test helped us determine if the observed frequencies across different categories were significantly different from each other, assuming that the expected frequency for each category should roughly be the same if they were randomly distributed.

In Aim 2, we examined potential gender-based disparities in the distribution of mass shooting fatalities across locations by stratifying fatalities within each age group (children, older children and teens, and adults) by gender (male or female). We then employed a Chi-square test of independence to assess whether a statistically significant association existed between location and gender within each age group. However, recognizing the potential limitations of Chi-square tests when dealing with small sample sizes within certain categories, we utilized Fisher’s exact test for more accurate results in such instances.

In Aim 3, we investigated the potential association between fatality location and domestic violence, we stratified fatalities within each age group by domestic violence status, classifying each fatality as either “domestic violence related” or “non-domestic violence related.” We then used Chi-square tests to determine the statistical significance of the association, with Fisher’s exact test being employed for categories with small sample sizes to ensure accurate results.

## Results

### Where are child, teen, and adult mass shooting fatalities occurring?

Over the ten-year period, we observed a total of 141 mass shooting fatalities ages 0–9. The majority of fatalities in this age group occurred in a home (89%), over 22 times the proportion that occurred in the second most common location, school (4%) (Table 1). We detected a statistically significant deviation from equal frequencies across all fatality locations for ages 0–9, indicating that fatalities in this age group were not evenly distributed across locations (Chi-square test, *p* < 0.001). When we compared home location with all non-home locations combined into one category, we found statistically significant evidence that fatalities in this age group were disproportionately concentrated in the “home” category compared with all other locations combined (Chi-square test, *p* < 0.001).

A higher number of mass shooting fatalities ages 10–17 (*n* = 167) occurred over the ten-year study period. Home was again found to be the most common setting, accounting for 62% of all fatalities in this age group (Table 2). The second-most common location, school, accounted for 25% of fatalities, less than half the fatalities that occurred in a home. The next most common locations were multiple locations, place of worship, and business, accounting for 5%, 2%, and 2% of fatalities in this age group, respectively. We detected a statistically significant deviation from equal frequencies across all fatality locations for ages 10–17, indicating that fatalities were not evenly distributed across locations (Chi-square test, *p* < 0.001). When we compared home versus all non-home locations combined for this age group, we found statistically significant evidence that fatalities are disproportionately concentrated in the “home” category compared with all other locations combined (Chi-squared test, *p* < 0.001).

For adult mass shooting fatalities ages 18 or older (*n* = 1,123), we again found home to be the most frequent location, accounting for 44% of all adult fatalities and almost four times the proportion that occurred in the second most common locations, place of business (12%) or spree shootings in multiple locations (12%) (Table 3). We found a statistically significant deviation from equal frequencies across all fatality locations, indicating that fatalities are not evenly distributed across locations for adults (Chi-square test, *p* < 0.001). Our analysis revealed a statistically significantly higher likelihood of adult fatalities occurring within home settings compared to all other locations combined (Chi-squared test, *p* < 0.001). When we examined young adult fatalities (ages 18–25) separately, we found that a higher proportion of mass shooting fatalities in this age group (53%) occurred in home-based shootings than adult fatalities ages 26 or older (42%). Younger adult mass shooting fatalities occurred more frequently in bars or nightclubs (14%) than older adults (6%), while older adult fatalities occurred more frequently in workplaces or businesses that were not bars or restaurants (14%) than younger adults (6%) (Table 3). We found statistically significant evidence that fatalities are not evenly distributed across locations for both adult age groups (Chi-square test, *p* < 0.001), and that adult fatalities in both age groups had a higher likelihood of occurring in the home compared to all other locations combined (Chi-squared test, *p* < 0.001).

### How does location of shooting relate to gender of fatalities?

It is noteworthy that home was the most frequent location of mass shooting fatality for both males and females across all age groups. While no significant gender differences were observed in the location of fatality among children and older children and teens, a higher proportion of adult (ages 18 or older) female fatalities (50%) occurred at home compared to adult males (40%) (Table 3) (Fisher’s exact test, *p* < 0.05).

### How does domestic violence relate to fatalities across locations?

Mass shooting fatalities occurred slightly less frequently in domestic violence related events (47%) than in non-domestic violence related events (54%) overall. These percentages, though, varied significantly across locations. Home, the most frequent location of fatalities across all age groups, exhibited a notably high proportion of domestic violence related fatalities (70%) than non-domestic violence related fatalities (31%) (Table 4). Among events that occurred at home but were not domestic violence-related, circumstances included drug or property disputes and robbery. Places of worship (56%) and locations involving spree shootings (56%) also made up a substantial proportion of domestic violence related shooting fatalities. Conversely, businesses, schools, and bars/nightclubs were more likely to be associated with fatalities from non-domestic violence related shootings. We found a statistically significant association between location and whether a mass shooting fatality occurred in a DV-related event (Table 4) (Fisher’s exact test, *p* < 0.001). When we compared home location with all other locations, we found statistically significant evidence that fatalities from DV-related events had a higher likelihood of occurring in the home compared to all but one other location (Chi-squared test, *p* ≤ 0.01).

Results from our sensitivity analysis, which excluded fatalities from unsolved mass shootings, were consistent with the main analysis (Appendix A).

## Discussion

Home-based events accounted for a substantial proportion of mass shooting fatalities, with over half (50%) of all fatalities across all ages and genders occurring within residential settings. In contrast, businesses or workplaces constituted the second most common single location but accounted for just 10% of all fatalities. These findings strongly suggest that mass shootings in private residences must be treated as a central focus of prevention efforts. Excluding home-based events from analyses of mass shootings risks excluding over half of fatalities, undermining efforts to develop effective prevention strategies. While public places like schools and workplaces receive most media focus, funding, and preparedness effort, the highest-risk setting—the home—is often overlooked. This skewed focus may also distort communities’ and policymakers’ perceptions of risk and may reduce the likelihood that families get the societal support needed to reduce risk of violence in the home.

Focusing on public mass shootings also obscures the disproportionate effect of home-based shootings on women and youth under 18. Home-based shootings resulted in over 20 times the fatalities among children aged 0–9 and more than twice the fatalities among youth aged 10–17 compared to school shootings, yet public discourse has overwhelmingly centered on school-based events. While school preparedness remains important, our findings call for an urgent expansion of prevention strategies to protect young people from fatal violence in the home, where they are far more likely to die in mass shootings. Similarly, markedly greater numbers of adult female (50%) fatalities occurred in a home than in a place of business (12%), yet far more public attention has been paid to workplace mass shooting events. Ensuring these disparities are captured in research and public attention may lead to the development of more effective prevention efforts.

Our finding that 47% of mass shooting fatalities occurred in domestic violence related events supports a growing body of evidence in the literature that domestic violence is a major driver of mass shootings and demonstrates a critical need for interventions addressing mass shootings related to domestic violence. However, our data also show that almost a third (30%) of mass shooting fatalities that occurred in a home did not occur in a domestic violence-related event, demonstrating a need for broader interventions that extend beyond domestic violence prevention alone. These findings highlight the importance of regular monitoring of all mass shooting events across both public and private settings and characterizing them with precision to inform effective intervention strategies. Conflating “home-based” and “domestic violence-related” events risks misinforming both public understanding and public health and policy intervention design.

While a higher proportion of adult female mass shooting fatalities than adult male mass shooting fatalities occurred in home-based mass shootings, our finding that 40% of adult male mass shooting fatalities also occurred in the home. This points to the home as a critical but under-recognized setting for prevention for all ages and genders.

### Limitations

GVA was used for this analysis due to its inclusion of all events in which four or more individuals were injured, excluding the perpetrator, in all U.S. states since 2014, and because of its inclusion of fatality age and gender as variables. However, GVA does not include fatality race, ethnicity, or other demographic information, nor was this information available in much of the news coverage analyzed. To better understand who is affected by mass shootings that occurred in different locations, further studies should aim to include more fatality demographic information.

Of the fatalities in this sample, 75 occurred in unsolved events, for which there was not sufficient available information to determine event relationship to domestic violence. These fatalities account for 4% of the full sample. As discussed under methods, this subgroup was treated as fatalities from non-domestic violence related events in this analysis. We expect that this decision resulted in an undercount of the proportion of fatalities that occurred in domestic violence related events.

In this analysis, “home” includes all mass shooting fatalities that occurred in private residences, such as houses or apartments. For individuals with unstable housing, locations like cars or streets may have been “home” shootings but were not counted as such in this analysis. Similarly, spree shootings spanning multiple locations may have included fatalities that occurred in homes, but because the exact location of each fatality could not always be determined, these were categorized as “multiple locations.” These limitations likely resulted in an undercount of mass shooting fatalities that occurred in homes, meaning the reported findings may underestimate the true proportion, further reinforcing the conclusions.

### Implications

The findings of this study suggest multiple concrete avenues for enhancing prevention. First, reframing mass shooting research and prevention efforts to include the home as a high-risk setting may lead to more widespread integration of home-based risk factors into mass shooting research, media narratives, and prevention efforts by communities and policymakers. Second, given the strong overlap between domestic violence and home-based shootings, improved coverage and enforcement of state laws that remove firearms from perpetrators of domestic abuse may help reduce mass shooting fatalities [18, 25]. Improving access to safety planning, housing, and legal supports for survivors of previous incidents may also help prevent subsequent DV incidents from escalating to mass violence. Third, given the finding that almost a third of home-based fatalities did not occur in DV-related events, approaches that extend beyond DV such as mental health outreach and trauma-informed care, crisis intervention programs, safe firearm storage campaigns, or Extreme Risk Protection Order (ERPO) policies may also be effective at reducing fatality from home-based shootings. And lastly, resources and policy efforts that disproportionately focused on schools and workplaces should be rebalanced to address risks where fatalities most commonly occur.

## Conclusion

This study is the first to comprehensively examine the intersection of mass shooting location, victim age and gender, and event relationship to domestic violence. In doing so, it represents a crucial step in recognizing mass shootings as a multifaceted public health concern and contributes critical evidence to guide the development of nuanced, location-sensitive prevention strategies. By demonstrating that home-based mass shootings account for a higher proportion of fatalities than any other location, this study challenges prevailing narratives around mass shootings and offers a foundation for more targeted, inclusive, and effective public health responses. Future research should explore whether interventions and policies that address domestic violence, such as policies that remove firearms from perpetrators of domestic violence or upstream efforts aimed to prevent domestic violence before it occurs, could be effective strategies to reduce fatalities from home-based mass shootings and fatalities from mass shootings overall.

**Table 1:** Location of mass shooting fatalities ages 0–9 by fatality gender (*N* = 141)

Location	Female 0–9	Male 0–9	Total
	N (*%*)	N (*%*)	N (*%*)
Home	50 (*84.7*)	75 (*91.5*)	125 (*88.7*)
School	4 (*6.8*)	2 (*2.4*)	6 (*4.3*)
Place of worship	3 (*5.1*)	1 (*1.*2)	4 (*2.8*)
Other**	2 (*3.4*)	2 (*2.4*)	4 (*2.8*)
Business	0 (*0.0*)	1 (*1.2*)	1 (*0.7*)
Festival	0 (*0.0*)	0 (*0.0*)	0 (*0.0*)
Multiple***	0 (*0.0*)	1 (*1.2*)	1 (*0.7*)
Bar	0 (*0.0*)	0 (*0.0*)	0 (*0.0*)
Street	0 (*0.0*)	0 (*0.0*)	0 (*0.0*)
Car	0 (*0.0*)	0 (*0.0*)	0 (*0.0*)
Restaurant	0 (*0.0*)	0 (*0.0*)	0 (*0.0*)
Total	59 (*100.0*)	82 (*100.0*)	141 (*100.0*)

** “Other” combines locations with *n* < 10 in the full sample. These include group home, airport, military base, hospital, shopping mall, campground, abandoned building, dance studio, motel, and non-residential farm

*** “Multiple locations” denotes spree shooting that occurred in multiple locations. Some spree shootings included residence, business, street, or car locations, but are not included in counts for those locations

**Table 2:** Location of mass shooting fatalities ages 10–17 by fatality gender (*N* = 167)

Location	Female 10–17	Male 10–17	Total
	N (*%*)	N (*%*)	N (*%*)
Home	61 (*62.9*)	42 (*60.0*)	103 (*61.7*)
School	26 (*26.8*)	17 (*24.3*)	43 (*25.7*)
Multiple**	3 (*3.1*)	6 (*8.6*)	9 (*5.4*)
Place of worship	3 (*3.1*)	1 (*1.4*)	4 (*2.4*)
Business	1 (*1.0*)	2 (*2.9*)	3 (*1.8*)
Other*	1 (*1.0*)	1 (*1.4*)	2 (*1.2*)
Festival	0 (*0.0*)	0 (*0.0*)	0 (*0.0*)
Bar	1 (*1.0*)	0 (*0.0*)	1 (*0.6*)
Street	0 (*0.0*)	1 (*1.4*)	1 (*0.6*)
Car	1 (*1.0*)	0 (*0.0*)	1 (*0.6*)
Restaurant	0 (*0.0*)	0 (*0.0*)	0 (*0.0*)
Total	97 (*100.0*)	70 (*100.0*)	167 (*100.0*)

* “Other” combines locations with *n* < 10 in the full sample. These include group home, airport, military base, hospital, shopping mall, campground, abandoned building, dance studio, motel, and non-residential farm

*** “Multiple locations” denotes spree shooting that occurred in multiple locations. Some spree shootings included residence, business, street, or car locations, but are not included in counts for those locations

**Table 3:** Location of adult mass shooting fatalities by fatality gender (*N* = 1,123)

Location	Adult Female	Adult Male	Total
	N (*%*)	N (*%*)	N (*%*)
*All adult (18+)*			
Home	243 (*49.5*)	254 (*40.2*)	497 (*44.3*)
Multiple**	67 (*13.6*)	70 (*11.1*)	137 (*12.2*)
Business	57 (*11.6*)	82 (*13.0*)	139 (*12.4*)
Bar	18 (*3.7*)	68 (*10.8*)	86 (*7.7*)
Other*	25 (*5.1*)	44 (*7.0*)	69 (*6.1*)
Festival	37 (*7.5*)	38 (*6.0*)	75 (*6.7*)
Place of worship	20 (*4.1*)	20 *(3.2*)	40 (*3.6*)
School	14 (*2.9*)	11 (1.7)	25 (*2.2*)
Car	4 (*0.8*)	15 (*2.4*)	19 (*1.7*)
Street	4 (*0.8*)	15 (*2.4*)	19 (*1.7*)
Restaurant	2 (*0.4*)	15 (*2.4*)	17 (*1.5*)
Total	491 (*100.0*)	632 (*100.0*)	1123 (*100.0*)
*Adult 18–25*			
Home	49 (*58.3*)	77 (*50.7*)	126 (*53.4*)
Multiple**	4 (*4.8*)	9 (*5.9*)	13 (*5.5*)
Business	6 (*7.1*)	7 (*4.6*)	13 (*5.5*)
Bar	8 (*9.5*)	26 (*17.1*)	34 (*14.4*)
Other*	2 (*2.4*)	8 (*5.3*)	10 (*4.2*)
Festival	4 (*4.8*)	4 (*2.6*)	8 (*3.4*)
Place of worship	0 (*0.0*)	0 (*0.0*)	0 (*0.0*)
School	6 (*7.1*)	4 (*2.6*)	10 (*4.2*)
Car	1 (*1.2*)	10 (*6.6*)	11 (*4.7*)
Street	2 (*2.4*)	4 (*2.6*)	6 (*2.5*)
Restaurant	2 (*2.4*)	3 (*2.0*)	5 (*2.1*)
Total	84 (*100.0*)	152 (*100.0*)	236 (*100.0*)
*Adult 26+*			
Home	194 (*47.7*)	177 (*36.9*)	371 (*41.8*)
Multiple**	63 (*15.5*)	61 (*12.7*)	124 (*14.0*)
Business	51 (*12.5*)	75 (*15.6*)	126 (*14.2*)
Bar	10 (*2.5*)	42 (*8.8*)	52 (*5.9*)
Other*	23 (*5.7*)	36 (*7.5*)	59 (*6.7*)
Festival	33 (*8.1*)	34 (*7.1*)	67 (*7.6*)
Place of worship	20 (*4.9*)	20 (*4.2*)	40 (*4.5*)
School	8 (*2.0*)	7 (*1.5*)	15 (*1.7*)
Car	3 (*0.7*)	5 (*1.0*)	8 (*0.9*)
Street	2 (*0.5*)	11 (*2.3*)	13 (*1.5*)
Restaurant	0 (*0.0*)	12 (*2.5*)	12 (*1.4*)
Total	407 (*100.0*)	480 (*100.0*)	887 (*100.0*)

* “Other” combines locations with *n* < 10 in the full sample. These include group home, airport, military base, hospital, shopping mall, campground, abandoned building, dance studio, motel, and non-residential farm

** “Multiple locations” denotes spree shooting that occurred in multiple locations. Some spree shootings included residence, business, street, or car locations, but are not included in counts for those locations

**Table 4:** Location of mass shooting fatalities by event relationship to domestic violence (DV) (*N* = 1,464)

Location	Not DV-related*	DV-related	Total	*P* Value^+^
	N (*%*)	N (*%*)	N (*%*)	
Home/residence	226 (*30.6)*	513 (*69.4*)	739 (*100.0*)	
Business	140 (*94.6*)	8 (*5.4*)	148 (*100.0*)	< 0.001
Bar/Nightclub	87 (*100.0*)	0 (*0.0*)	87 (*100.0*)	< 0.001
Festival/Parade	75 (*100.0*)	0 (*0.0*)	75 (*100.0*)	< 0.001
School	50 (*66.7*)	25 (*33.3*)	75 (*100.0*)	< 0.001
Place of worship	21 (*43.8*)	27 (*56.3*)	48 (*100.0*)	0.057
Restaurant	21 (*100.0*)	0 (*0.0*)	21 (*100.0*)	< 0.001
Car	16 (*80.0*)	4 (*20.0*)	20 (*100.0*)	< 0.001
Street	20 (*100.0*)	0 (*0.0*)	20 (*100.0*)	< 0.001
Conference Center	14 (*100.0*)	0 (*0.0*)	14 (*100.0*)	< 0.001
Other**	57 (*93.4*)	4 (*6.6)*	61 (*100.0*)	< 0.001
Multiple Locations***	69 (*44.2*)	87 (*55.8*)	156 (*100.0*)	0.001
Total	796 (*54.4*)	668 (4*6.6*)	1464 (*100.0*)	

* Includes fatalities from events with indeterminant relationship to domestic violence (*N* = 75)

^+^*P* values indicate the results of Pearson Chi-squared tests, comparing proportion of fatalities related to domestic violence occurring in each location with those occurring in home/residence

**“Other” combines of locations with *n* < 10 in the full sample. These include group home, airport, military base, hospital, shopping mall, campground, abandoned building, dance studio, motel, and non-residential farm

*** “Multiple locations” denotes spree shooting that occurred in multiple locations. Some spree shootings included residence, business, street, or car locations, but are not included in counts for those locations

## Electronic supplementary material

Supplementary Material 1

## Data Availability

The datasets used and/or analyzed during the current study are available from the corresponding author upon reasonable request.

## Abbreviations

DV: Domestic violence
GVA: Gun Violence Archive
