# Supplementary material for: The overlooked role of the home in mass shooting fatalities
Source: Inj Epidemiol. 2025 Jul 21;12:44. doi: 10.1186/s40621-025-00602-z (PMC12278596; doi:10.1186/s40621-025-00602-z)
Supplement: Supplementary file 1 — Supplementary Material 1 [file 40621_2025_602_MOESM1_ESM.docx]

**Appendix A**

When mass shooting fatalities from unsolved events were omitted from analyses, we found a greater percentage of total fatalities occurred in domestic violence (DV)-related events (48%) than in our main analysis (47%). Among fatalities from home-based shootings specifically, we also found a higher proportion of mass shooting fatalities occurred in DV-related events (73%) when fatalities from unsolved events were omitted than in our main analyses (69%). As in our main analysis, we found a statistically significant association between location and whether a mass shooting fatality occurred in a DV-related event (Fisher's exact test, p<0.001) and when we compared home location with all other locations, we found statistically significant evidence that fatalities from DV-related events had a higher likelihood of occurring in the home compared to all other locations (Chi-squared test, p­­<0.01).

**Table A1**: Location of mass shooting fatality by event relationship to domestic violence (DV), excluding fatalities from unsolved events (N=1,389)

|  | Not DV-related | DV-related | Total |  |
| --- | --- | --- | --- | --- |
| Location | N (*%*) | N (*%*) | N (*%*) | P Value^+^ |
| Home/Residence | 186 (*26.6)* | 513 (*73.4*) | 699 (*100.0*) |  |
| Business | 133 (*94.3*) | 8 (*5.7*) | 141 (*100.0*) | <0.001 |
| Bar/Nightclub | 87 (*100.0*) | 0 (*0.0*) | 87 (*100.0*) | <0.001 |
| Festival/Parade | 71 (*100.0*) | 0 (*0.0*) | 71 ((*100.0*) | <0.001 |
| School | 50 (*66.7*) | 25 (*33.3*) | 75 (*100.0*) | <0.001 |
| Place of worship | 21 (*43.8*) | 27 (*56.3*) | 48 (*100.0*) | 0.010 |
| Restaurant | 17 (*100.0*) | 0 (*0.0*) | 17 (*100.0*) | <0.001 |
| Car | 8 (*66.7*) | 4 (*33.3*) | 12 (*100.0*) | 0.002 |
| Street | 12 (*100.0*) | 0 (*0.0*) | 12 (*100.0*) | <0.001 |
| Conference Center | 14 (*100.0*) | 0 (*0.0*) | 14 (*100.0*) | <0.001 |
| Other* | 53 (*93.0*) | 4 (*7.0)* | 57 (*100.0*) | <0.001 |
| Multiple Locations** | 69 (*44.2*) | 87 (*55.8*) | 156 (*100.0*) | <0.001 |
| Total | 721 (*51.9*) | 668 (*48.1*) | 1389 (*100.0*) |  |

^+^P values indicate the results of Pearson Chi-squared tests, comparing proportion of fatalities related to domestic violence occurring in each location with those occurring in home/residence

*“Other” combines of locations with n<10 in the full sample. These include group home, airport, military base, hospital, shopping mall, campground, abandoned building, dance studio, motel, and non-residential farm

** “Multiple locations” denotes spree shooting that occurred in multiple locations. Some spree shootings included residence, business, street, or car locations, but are not included in counts for those locations
